# Sex as predictor of employment at 5 years follow-up in First Episode Psychosis

**DOI:** 10.1192/j.eurpsy.2024.794

**Published:** 2024-08-27

**Authors:** A. Toll, E. Pechuan, T. Legido, D. Berge, A. Manzano, K. El Abidi, V. Perez-Sola, A. Mane

**Affiliations:** ^1^Institut de Salud Mental, Hospital del Mar, Barcelona, Spain

## Abstract

**Introduction:**

Despite considerable growth in the last years in treatments and research in first episode psychosis (FEP), little attention has been given to the priorities of these young people, in particular, gaining employment. For most people, work is a normal part of everyday life and can be considered one of the most important factors in promoting recovery and social inclusion. Nevertheless, these patients show low employment rates (varying from 23% to 65%) since the beginning of the psychotic symptoms and even after their contact with mental health services. But, although completing education and access to employment is a critical part for the recovery of these patients, few studies have focused on this outcome.

**Objectives:**

To determine the employment rate and its possible predictor factors in a FEP sample after 5 years follow – up.

**Methods:**

190 FEP treated between June 2010 and July 2013 at the ETEP Program at Hospital del Mar were included. Inclusion criteria were: 1) age 18-35 years; 2) fulfillment of DSM-IV-TR criteria for brief psychotic disorder, schizophreniform disorder, schizophrenia or unspecified psychosis; 3) no previous history of severe neurological medical conditions or severe traumatic brain injury; 4) IQ level < 80, and 5) no substance abuse or dependence disorders except for cannabis and/or nicotine use. All patients underwent an assessment at baseline including sociodemographic and clinical variables (substance use, DUP, PANSS and GAF). Moreover, employment status has recorded at 5 years follow – up as dichotomyc variable (being employment defined as having either a full-/part-time job, being a student at school or university, or being involved in a study/training program). SPSS program was used for statistical analyzes.

**Results:**

In our FEP sample, the employment rate was 34.2%. We observed significative differences in sex (p = 0.013), cannabis use (p = 0.022) and GAF scores (p = 0.016) between un/employed patients. Nevertheless, in the logistic regression model (ENTER METHOD) only female sex remained as predictor of higher employment rate (95% CI 1.13 to 4.85; p = 0.022) at 5 years follow – up.

**Conclusions:**

Our results suggest that females with a FEP have a better outcome in terms of employment rates, consistent with some previous studies. Some authors suggest that it could be explained by the fact that female patients used to have shorter DUP or more affective symptomatology, which has been also related to a better outcome. Nevertheless, we did not find any differences in these other variables in our sample. Employment not only provides financial independence but also structure and purpose, opportunities for socializing and developing new relationships, a sense of identity, self-worth and meaning in life. Thus, given its importance in FEP functional recovery, more studies in this field are needed to improve patients vocational achievements and determine which specific approaches would each of them need.

**Disclosure of Interest:**

None Declared

